# The Burnout Syndrome in Medical Academia: Psychometric Properties of the Serbian Version of the Maslach Burnout Inventory—Educators Survey

**DOI:** 10.3390/ijerph17165658

**Published:** 2020-08-05

**Authors:** Marijana Vukmirovic, Nina Rajovic, Vedrana Pavlovic, Srdjan Masic, Momcilo Mirkovic, Radica Tasic, Simona Randjelovic, Danka Mostic, Igor Velickovic, Emilija Nestorovic, Petar Milcanovic, Dejana Stanisavljevic, Natasa Milic

**Affiliations:** 1 Center for Informatics and Biostatistics, Belgrade Public Health Institute, 11000 Belgrade, Serbia; jewdjiq@gmail.com; 2 Institute for Medical Statistics and Informatics, Faculty of Medicine, University of Belgrade, 11000 Belgrade, Serbia; nina.rajovic@med.bg.ac.rs (N.R.); vedrana.pavlovic@med.bg.ac.rs (V.P.); petar.milcanovic@gmail.com (P.M.); dejana.stanisavljevic@med.bg.ac.rs (D.S.); 3 Department for Public Health, Faculty of Medicine, University of East Sarajevo, 73300 East Sarajevo, Bosnia and Herzegovina; srdjan.masic@ues.rs.ba; 4 Institute of Social Medicine, Faculty of Medicine, University of Pristina, 38220 Kosovska Mitrovica, Serbia; momcilomirkovic76@gmail.com; 5 Medical School, Academy of Vocational Studies Belgrade, 11000 Belgrade, Serbia; radica.logoped@gmail.com (R.T.); velickovicigor@live.com (I.V.); 6 Clinical Center of Serbia, 11000 Belgrade, Serbia; simonarandjelovic@gmail.com (S.R.); mosticd@gmail.com (D.M.); emanestor@gmail.com (E.N.); 7 Department of Internal Medicine, Division of Nephrology and Hypertension, Mayo Clinic, Rochester, MN 55902, USA

**Keywords:** burnout, MBI-ES, educators, medicine, psychometric properties, personality, career change, work abroad

## Abstract

The aim of this study was to assess the psychometric properties of the Maslach Burnout Inventory—Educators Survey (MBI-ES). The presence of burnout syndrome, its relationship with personality traits, intention to change career and work abroad were assessed in a cross-sectional multi-center trial conducted among educators at three medical faculties in the Western Balkans during 2019. Translation and cultural adaptation were made based on internationally accepted principles. Personality traits were assessed by the Big Five Plus Two questionnaire. In total, 246 medical faculty members, predominantly females (61%), were enrolled. The three-factor structure of the MBI-ES questionnaire (exhaustion, cynicism, and professional efficacy) was validated. Analysis of internal consistency yielded a Cronbach’s alpha of 0.785, indicating scale reliability. The majority of respondents (85.6%) reported moderate level of burnout. Aggressiveness, neuroticism, and negative valence were associated with emotional exhaustion and depersonalization, while extraversion, conscientiousness, openness, and positive valence correlated with personal accomplishment. Emotional exhaustion and depersonalization in a multivariate regression model were significantly associated with intentions to change career and work abroad (*p* < 0.05). The present study provided evidence for the appropriate metric properties of the Serbian version of MBI-ES. Presence of burnout syndrome, which was identified as a common problem in medical academia, and directly linked to personality traits, affected intention to career change and work abroad.

## 1. Introduction

Burnout is a syndrome related to work and is usually recognized in professions where cooperation and personal engagement with others is high [1]. There is a lack of studies assessing burnout among the university faculty, with even less conducted on medical faculty, who work under pressure to extend their role beyond clinical work to the educational process [2,3]. Medical faculty are encountering considerable stress as a result of the liability and administration associated with patient care, teaching, and research [4].

The gold standard for measuring burnout syndrome severity is the Maslach Burnout Inventory (MBI) questionnaire originally developed in the 1980s [1]. Burnout syndrome among educators is defined by high levels of emotional exhaustion, depersonalization, and reduced sense of personal accomplishment at work [1,5]. According to Maslach et al., emotional exhaustion is the central point of burnout, representing feelings of being emotionally overextended and exhausted [1,5]. Exhaustion is the most extensively reported as a mandatory criterion for burnout syndrome. Depersonalization is the outcome of negative, uncaring, and contemptuous attitudes towards students. High level of depersonalization presents educators attempt to separate emotional and rational by ignoring the student’s nature, personality, and appearance. The third aspect of burnout syndrome—personal accomplishment, refers to evaluation of achievements at work. Maslach et al. proposed that reduced personal accomplishment is related to inefficacy at work and negative evaluation of oneself [1,5].

According to the recent research, personal traits should not be neglected when it comes to burnout syndrome [6]. Having a certain personality trait may self-select an individual into extremely stressful and burnout-conducive professions. Additionally, certain personality traits may predispose employees to experiencing and coping with stressors more intensely, thereby subsequently eliciting burnout and contributing to the onset of burnout syndrome over time [7,8]. In response to the need for the personality assessment, the Big Five Plus Two (Big 5 + 2) questionnaire is commonly used [9]. Researchers have linked burnout to neuroticism more frequently than to any other of the Big Five factors of personality [10]. A tendency to interpret events negatively, having distressing emotions, and associated cognitive and behavioral traits characterizes neuroticism. In general, people who are excessive in neuroticism tend to set extremely high goals for themselves and underestimate their own achievements. Plausibly, this predisposition could negatively impact burnout levels over time [11].

Conscientiousness refers to the tendency to be thorough, organized, and reliable, and if inhabited, predisposes individuals to handle stress more efficiently. The conscientious individual’s self-discipline and persistency could presumably act as a buffer against burnout [12,13]. In the study of Piedmont and Deary et al. [14,15,16], a positive relationship between conscientiousness and personal accomplishment was found. In addition, extraversion is related with a tendency to reappraise problems positively and to be optimistic [17]. Enthusiastic temperament of extraverts focus them on the positive and beneficial side of their experiences [18]. It is therefore not surprising that a negative relationship between extraversion and burnout was found in some studies [14,19,20]. More studies relating personality factors to burnout may provide insights into whether burnout is a social occurrence or is associated to individual variability, and more importantly, may help to identify individuals who are prone to developing burnout.

Medical faculty have a key position in medical community. For residents, students, and colleagues, they are representative inspiration, with overall responsibility for management assignment beside research and educational activities at the same time [2]. There are many reasons for an intense and lasting interest in the presence of burnout syndrome in medical academia. In Serbia, there is a high intention to leave the country and work abroad, especially among healthcare professionals [21,22]. Estimates are that around 10,000 doctors left Serbia to find work abroad in the past 20 years. In addition, reports from the Serbian Medical Chamber state that around 800 doctors annually ask for a certificate of good practice, a document necessary for employment in certain countries. No institution, however, reports the official estimates of emigration status of licensed medical personnel and this substantial issue remains understudied. In line with our assumption, burnout of medical faculty and their willingness to work abroad and career change are in potential correlation. The aim of this study was (1) to assess the psychometric properties of the MBI—Educators Survey (MBI-ES) questionnaire, (2) to assess the presence of burnout in medical academia, (3) its relationship with personality traits, and (4) intention to change career and work abroad.

## 2. Materials and Methods

### 2.1. Design

This was a cross-sectional multi-center study conducted at the Medical Faculties of the University of Belgrade, East Sarajevo and Pristina among professors and assistant professors during the 2018–2019 school year. Participation in research was volunteer-based and complete confidentiality was maintained for respondents. The ethical approval (no.: 01-1098) was obtained from the Ethics Committees of all Medical Faculties involved in the research. Demographic and occupational characteristics of the study sample were collected at the beginning of the study.

### 2.2. Instrument

The MBI-ES questionnaire was used to measure burnout syndrome. The permission was obtained from the copyright owner (Sinapsa Edicija doo, a member of the Naklada Slap group from Zagreb, contract no.: May 2017). Translation and cultural adaptation of the research instrument were made on the basis of internationally accepted principles [23]. The original version of the questionnaire was translated into Serbian by two independent translators whose mother tongue is Serbian. In order to provide a translation that closely resembles the original instrument, one translator was aware of the concepts the questionnaire intend to measure, while another one was naive, aiming to produce the translation in which the subtle differences may be found. Discrepancies were discussed and resolved between the original translators. To ensure the accuracy of the translation, in the next step, the instrument was back-translated by two independent native speaking English professors fluent in Serbian. To avoid bias, back-translators were not aware of the concepts the questionnaire intended to measure. The original version was compared to the back-translated version and differences were resolved through consensus by members of an expert committee. Members of the committee included methodology experts who were familiar with the construct of interest and both the forward and backward translators. Clarity, comprehensibility and acceptability of the Serbian version of questionnaire were than evaluated by 20 university professors. Modifications were done based on participants’ feedback before the final version was distributed.

The MBI-ES consists of three subscales: emotional exhaustion, depersonalization, and personal accomplishment. In total, there are 22 items: 9 questions assess emotional exhaustion, 5 depersonalization, and 8 questions evaluate personal accomplishment. Responses to each item are scored on a 6-point Likert scale. High values of emotional exhaustion and depersonalization subscales correspond to high level of burnout. Low values of professional accomplishment subscale point to a strong experience of combustion at work. Final results were categorized as low, moderate, and high burnout in medical academia.

Personality traits were assessed by Big 5 + 2 Questionnaire. This instrument is comprised of seven traits: neuroticism, extraversion, openness, conscientiousness, aggression, and positive and negative valence. A shorter 70-item version was used to measure each of the seven scales. A five-step Likert response scale ranging from “strongly disagree” (1) to “strongly agree” was applied (5). Additionally, data regarding intention to work abroad or career change were collected. Participants were asked if they planned to change their current job or to work abroad or were not thinking about that at all.

### 2.3. Data Analysis

The descriptive statistics, including means, medians, and standard deviations for numerical variables, and numbers and percentages for categorical variables, were used to characterize the study sample. The sample size estimation was based on the assumption needed to fulfill factor analysis use, set by Tabachnick and Fidell [24], where the minimum number of respondents must be 150, with at least 5 respondents for each item. To estimate the reliability of questionnaires, the Cronbach’s alpha coefficients (ranges from 0-1, the latter meaning perfect reliability) were calculated. Confirmatory factor analysis was conducted to assess the three-dimensional structure of MBI-ES. The chi-square test was used to test the absolute goodness-of-fit of the model. Additionally, three fit indices were estimated: comparative-fit index (CFI), Tucker-Lewis index (TLI), and the root mean square error of approximation (RMSEA). Values of CFI and TLI above 0.90 were considered adequate, whereas RMSEA values below 0.08 indicated an acceptable model fit. CFA was conducted using Amos 21 (IBM SPSS Inc., Chicago, IL, USA). Univariate and multivariate logistic regression analyses were used to determine factors related to intention to work abroad and career change. Significant variables from univariate analysis were included in multivariate regressions. In multivariate regression, with career change as an outcome, emotional exhaustion and depersonalization were used as independent variables. In multivariate regression, with intention to work abroad as an outcome, emotional exhaustion, depersonalization, and performing clinical work were used as independent variables. Results were expressed as B, Wald chi-square, odds ratios (OR), and their corresponding 95% confidence intervals (CI). In all analyses, the level of statistical significance was set at *p* ≤ 0.05. The statistical analysis was performed using IBM SPSS 21 (Chicago, IL, USA).

## 3. Results

### 3.1. Study Population

The Serbian versions of MBI-ES and Big 5 + 2 questionnaire were completed by 246 professors and assistant professors currently employed at three universities. The mean age of respondents was 46.1 ± 9.4 years, and most participants were female (61%). The median length of service was 19 years, ranging from 1 to 43. Almost half of the participants were employed at clinics (41.1%) and 29.8% held an executive position. In total, 11 percent of respondents had an intention to work abroad and 8.4% had an intention to change career (Table 1).

### 3.2. Psychometric Properties of the MBI-ES Questionnaire

**Figure 1:**
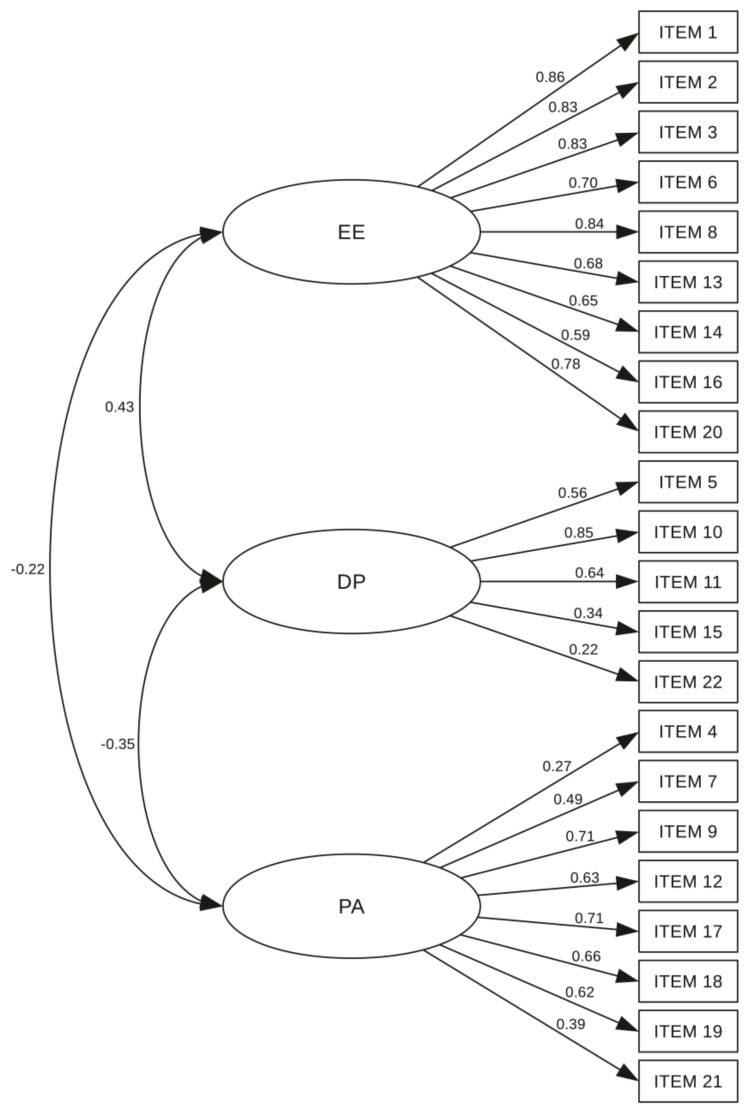
Standardized factor loadings for the Maslach Burnout Inventory—Educators Survey (MBI-ES) questionnaire in the Serbian language. EE, emotional exhaustion; DP, depersonalization; PA, personal accomplishment.

The three-factor structure of MBI-ES questionnaire has been validated with the maximum likelihood confirmatory analysis and the results demonstrated an acceptable level of model fit. The chi-square test rejected the three-dimensional model, as was expected due to the large sample size (χ^2^ = 596.133, df = 50, *p* < 0.001). The values for fit indices TLI (0.823) and CFI (0.842) were close to their cut-off criteria. The RMSEA value of 0.086 (CI 0.078–0.094) was around 0.08 indicating adequate fit. Standardized factor loadings were statistically significant and ranged from 0.22 to 0.86 (Figure 1). The analysis for internal consistency of the Serbian version of MBI-ES questionnaire yielded a Cronbach’s alpha of 0.785 for the entire scale, which indicated scale reliability. The alpha coefficient for the emotional exhaustion subscale was 0.93 (excellent reliability), while alpha coefficients for depersonalization and personal accomplishment subscales were estimated to be 0.618 (questionable) and 0.776 (acceptable), respectively (Table 2).

### 3.3. Presence of Burnout in Medical Academia

Maslach defines burnout as high emotional exhaustion, high depersonalization, and low personal accomplishment. In our study, the average subscale score for emotional exhaustion was 17.3 ± 11.8. Moderate to high level of emotional exhaustion was observed in 56.1% of medical faculty (Table 3). The average subscale score for depersonalization was 2.4 ± 3.3, and the scores were 65.2%, 25.8%, and 9.1% in the ranges of low, moderate, and high burnout, respectively. The average subscale score for personal accomplishment was 36.3 ± 7.9. According to these subscale results, most medical faculty had moderate and high levels of burnout (38.0% and 39.6%, respectively). Overall, only 3.2% of medical faculty met all three criteria for high burnout (high emotional exhaustion, high depersonalization, and low personal accomplishment) and 11.2% met all three criteria for low burnout (low emotional exhaustion, low depersonalization, and high personal accomplishment) revealing that 85.6% of medical faculty belong to moderate overall burnout category.

### 3.4. Burnout and Personality Traits in Medical Academia

Total Cronbach’s Alpha for the Big Five Plus Two questionnaire was 0.813, indicating good scale reliability. Mean subscale scores obtained using the Big 5 + 2 questionnaire for extraversion and positive valence were: 3.7 ± 0.7 and 3.1 ± 0.6, respectively. Low mean scores were found for aggressiveness (2.1 ± 0.7), neuroticism (2.0 ± 0.7), and negative valence (1.4 ± 0.6), while high average scores were found for openness and conscientiousness: 4.0 ± 0.6 and 4.0 ± 0.5, respectively. The results showed that aggressiveness, neuroticism, and negative valence were positively correlated with emotional exhaustion (*p* = 0.001, *p* < 0.001 and *p* = 0.004, respectively) and depersonalization (*p* < 0.001, *p* < 0.001 and *p* < 0.001, respectively), while negatively correlated with personal accomplishment subscale (*p* < 0.001, *p* < 0.001 and *p* < 0.001, respectively). Extraversion and conscientiousness were in significant negative correlation with emotional exhaustion (*p* < 0.001 and *p* = 0.012, respectively) and depersonalization (*p* < 0.001 and *p* = 0.001, respectively), and were positively correlated with personal accomplishment (*p* < 0.001 and *p* < 0.001, respectively). Openness was negatively correlated with depersonalization (*p* < 0.01), while openness and positive valence were positively correlated with personal accomplishment (*p* < 0.001 and *p* < 0.001, respectively). No significant relationship was found between emotional exhaustion and above mentioned two personal traits (Table 4).

### 3.5. Intention to Work Abroad and Career Change

Emotional exhaustion and depersonalization, in a multivariate regression model, were significantly associated with career change. Faculty with higher level of burnout, according to emotional exhaustion and depersonalization subscale values, were more willing to change career compared to those with lower results obtained for these two subscales. In a multivariate regression analysis, workplace and depersonalization were significantly associated with intention to work abroad. Faculty who work at clinics and those with higher level of burnout measured by depersonalization subscale had more intention to work abroad compared to those who work at institutes and those with lower depersonalization (Table 5).

## 4. Discussion

Aiming to assess the burnout syndrome among medical educators in a university environment, a Serbian version of the MBI-ES was developed and its psychometric properties were tested in the multicenter study. Adequate levels of instrument validity and reliability were confirmed inside the Serbian educational context. The main finding in the study was that the majority of educators reported moderate level of burnout in medical academia. The higher level of burnout was significantly associated with both career change and intention to work abroad.

For many years, burnout syndrome has been accepted as an occupational hazard for various people-oriented professions, with special emphasis on education, health care, and human services [25]. The experience of combustion at work, the way of its development and appearance, and its influence on the relations in which an individual participates may be understood as a reflection of basic human responses to the lack of fulfillment of set expectations at work. Studies using MBI as an instrument for measuring a burnout experience are important sources for a better understanding of the role of work in human life. To the best of our knowledge, a study focusing on burnout prevalence among medical faculty in the Western Balkans had never been published. Studies on this syndrome have been conducted in clinical settings [26,27] and in medical students [28]. In addition, other versions of the MBI have been validated in Serbian [29,30], but not the MBI-ES. In our study, 85.6% of respondents suffered from moderate level of burnout. Similar results were noted in a study conducted by Johns et al., who showed that 81% of academic chairs of otolaryngology have moderate degree of burnout on all subscales [31]. Cruz et al. revealed that 9% of ophthalmology department chairs experience high burnout characterized by high emotional exhaustion, high depersonalization, and low personal accomplishment [32]. Gabbe et al. found that 4% of chairs of obstetrics and gynecology from the United States and Puerto Rico satisfied all three subscales scores for high burnout [4].

Overall, personality traits were strongly correlated with burnout. One of the objectives of this study was to gain a better understanding of the association between personality traits and burnout risk among medical faculty. In their empirical study, Buhler and Land, presented the relationship between burnout syndrome and personality traits, answering to a constantly-posed issue: “why, under the same working conditions, one individual burns out, whereas another show no symptoms at all“. According to their study, an alternative explanation is the association of burnout with personality traits [33]. This study results, as well as the study of Piedmont, showed connection between neuroticism and burnout syndrome, where people who have higher values of neuroticism manifest higher emotional exhaustion and depersonalization domains of burnout syndrome [14]. Numerous authors certified a direct effect of neuroticism on psychological disorders [33,34,35]. Independent of type and time of the stress situation, neurotic personality mainly act neurotic, thus experiencing a lack of success in tasks they are set out to do. This type of person has difficulties when dealing with stressors, mostly in working fields where stress is inevitable [34]. Unlike neurotic temperament, extroverts pursue excitement and willfully take risks, searching for something new while acting impetuous and adventurous. It is a subject of debate in literature, whether this type of person is more tend to become emotionally exhausted or is more prone to have a sense of personal accomplishment. Opposite to our results, in a study by Bühler and Land [33], a positive correlation was found between extraversion and two domains of burnout syndrome: emotional exhaustion and depersonalization. Results obtained in our study correspond to results of Ghorpade, et al. [36], who measured personal traits with a mini-markers Big Five scale [37], in which extraversion, agreeableness, conscientiousness, neuroticism, and openness were the Big Five personality traits. They reported negative correlations between emotional exhaustion, and extroversion and agreeableness, while depersonalization was negatively correlated with conscientiousness and agreeableness. Personal accomplishment was positively correlated with openness, conscientiousness, agreeableness, and extroversion. The fact that personality traits are associated with the presence of burnout syndrome points to both theoretical facts and the results of numerous of studies, including ours.

Nowadays, an enduring problem is a worldwide healthcare personnel crisis. The healthcare system in Serbia is encountering serious challenges considering emigration of health workers, due to social, economic, professional, and individual factors. Opportunities for professional improvement, better earnings, and working conditions, as well as higher quality standards, are crucial motivators for migration of healthcare workers [38]. Since 2006, the Institute for Public Health of Serbia in cooperation with the Ministry of Health has been conducting a survey of satisfaction among employees in healthcare institutions in the Republic of Serbia. This national survey enables all employees in health institutions in the Republic of Serbia to express their views on opportunities for a professional development, equipment, time, earnings, interpersonal relationships, and cooperation with colleagues, as well as intention to change their job or work abroad. Results from the survey showed that 14.7% of employees would go to work abroad in the next 5 years [21]. In our study, 8.4% of respondents are planning to change career, while 11% have the intention to work abroad. According to Maslach et al., mismatch between working conditions and individual characteristics is a key predictor for burnout syndrome [39]. More importantly, burnout among healthcare workers has been related with expanded rates in the intent to leave medical profession as well as with higher rates of job turnover [40]. Several studies have associated burnout with one’s intention of leaving the profession [41,42]. Results of a survey conducted in Saxony, Germany showed that scores on subscales of emotional exhaustion and depersonalization were associated with an increased chance to wishing to go abroad for clinical work [40]. According to our results, the association between intention to change career and work abroad with two domains of burnout syndrome was found. In light of these results, burnout can be seen as a consequence of one’s dissatisfaction with working conditions, especially for those performing clinical work [22].

Prevention of burnout has proved to be deductible [43], therefore it could play a vast role in increasing the effectiveness of the healthcare system, as well as in decreasing the number of healthcare professionals emigrating from Serbia. Several strategies may be put in order to prevent and/or reduce burnout syndrome. The starting point for the prevention of burnout syndrome in medical academia may be set at a human resources staff level. They would conduct the interviews with job applicants to determine their personality traits in order to estimate compatibility for an adequate teaching and/or research position. The study published in 2017 suggested nine organization-level strategies to reduce burnout and promote engagement among physicians [44]. Some of them include developing and implementing targeted interventions (assemble team, focus groups) or promoting flexibility and work-life integrations [44]. Mindfulness-based stress reduction classes were also recommended, as they demonstrated significantly decreased burnout across a wide range of physicians [45]. According to Jha et al., the three recommendations may benefit physicians in preventing or decreasing burnout in the short, medium, and long term [46]. While better mental health services and improving operability have short and medium term effects, only structural solutions are proven to have strong and enduring impact. This means assignment of executive-level chief wellness officers to assess physician burnout and to create and conduct interventions in order to reduce burnout [46]. Many of the above mentioned measures are relatively inexpensive and could be a key part in the effectiveness of the healthcare system. Driven by the results of this study and a mountainous body of evidence on the causes and impacts of physician burnout on emigration, this paper is a wakeup call to implement strategies focusing on decreasing the number of healthcare professionals who intend to emigrate from Serbia and work abroad.

### Limitations and Future Lines of Research

Possible selection bias in the inclusion of surveyed members of medical academia may be present, leading to subsequent overrepresentation of both members who suffer from high levels of burnout and those who suffer less. This might be explained by an unwillingness to participate for different reasons, such as hesitation to answer candidly, despite full confidentiality and preservation, or as a sense of wasted time due to increased burden of highly demanding responsibilities. In addition, it should be mentioned that the reasons academics may have for wanting to change careers or work abroad may go beyond burnout and dissatisfaction with working conditions, which should be the objective of further research.

## 5. Conclusions

The present study provided evidence for the appropriate metric properties of the Serbian version of MBI-ES. Confirmatory factor analysis validated the three-factor structure of the scale. Burnout was identified as a common problem in medical academia and was associated with both career change and intention to work abroad. Personality traits were characteristics that affected the presence of burnout syndrome. While an exhaustive list of solutions to address burnout syndrome is beyond the scope of this paper, the recommendations presented here may be used as a starting point for creating a sustainable work environment in medical academia.

## Figures and Tables

**Table 1:** Demographic and occupational characteristics of the study sample.

Variable	*n* (%)
Gender	
Male	83 (39)
Female	130 (61)
Age, mean ± SD	46.1 ± 9.4
Length of service, median (range)	19 (1–43)
University	
University of Belgrade	128 (48.5)
University of East Sarajevo	35 (13.3)
University of Pristina/Kosovska Mitrovica	101 (38.3)
Workplace	
Clinic	102 (41.1)
Institute	146 (58.9)
Position	
Executive position	76 (29.8)
Non-executive position	179 (70.2)
Career change	13 (8.4)
Work abroad	17 (11.0)

**Table 2:** Reliability Statistics for MBI-ES and Big Five Plus Two (Big 5 + 2) questionnaire.

Questionnaire	Cronbach’s Alpha	Internal Consistency
MBI-ES		
Emotional Exhaustion	0.923	Excellent
Depersonalization	0.618	Questionable
Personal Accomplishment	0.776	Acceptable
Total	0.785	Acceptable
Big Five Plus Two		
Aggressiveness	0.857	Good
Extraversion	0.853	Good
Neuroticism	0.881	Good
Negative Valence	0.864	Good
Openness	0.846	Good
Positive Valence	0.852	Good
Conscientiousness	0.686	Questionable
Total	0.813	Good

**Table 3:** Burnout syndrome among educators in University of Belgrade, East Sarajevo Kosovska Mitrovica.

Domain	Mean ± SD	Low, *n* (%)	Moderate *n* (%)	High *n* (%)
Emotional Exhaustion	17.3 ± 11.8	116 (43.9)	68 (25.8)	80 (30.3)
Depersonalization	2.4 ± 3.3	172 (65.2)	68 (25.8)	24 (9.1)
Personal Accomplishment *	36.3 ± 7.9	56 (22.4)	95 (38.0)	99 (39.6)
Overall burnout ^#^		28 (11.2)	214 (85.6)	8 (3.2)

Notes: Cutoff for low/moderate/high burnout are as follows: emotional exhaustion: ≤13, low;14–23, moderate; ≥24 high. Depersonalization: ≤2, low; 3–8, moderate; ≥9, high. Accomplishment: ≥43, low; 36–42, moderate; ≤35 high. * The accomplishment subscale is interpreted in the opposite direction as the emotional exhaustion and depersonalization subscales. ^#^ High burnout: high emotional exhaustion, high depersonalization, and low personal accomplishment; low burnout: low emotional exhaustion, low depersonalization, and high personal accomplishment.

**Table 4:** Correlations between personality traits and burnout.

Domains	Mean ± SD	Emotional Exhaustion	Depersonalization	Personal Accomplishment
Aggressiveness	2.1 ± 0.7	0.202 **	0.333 **	−0.297 **
Extraversion	3.7 ± 0.7	−0.393 **	−0.413 **	0.475 **
Neuroticism	2.0 ± 0.7	0.437 **	0.360 **	−0.373 **
Negative Valence	1.4 ± 0.6	0.182 **	0.351 **	−0.311 **
Openness	4.0 ± 0.6	−0.090	−0.338 **	0.457 **
Positive Valence	3.1 ± 0.6	−0.099	−0.113	0.340 **
Conscientiousness	4.0 ± 0.5	−0.160 **	−0.220 **	0.403 **

** *p* < 0.01

**Table 5:** Multivariate logistic regression models with career change and work abroad as dependent variables.

Variables	*B*	Wald Chi-Square	*p*	OR	95% CI for OR	
					Lower	Upper
Career change						
Emotional Exhaustion	0.443	4.012	0.045	1.557	1.010	2.402
Depersonalization	1.353	10.633	0.001	1.311	1.114	1.542
Work abroad						
Emotional Exhaustion	0.408	3.809	0.051	1.504	1.000	2.266
Depersonalization	0.989	5.973	0.015	2.690	1.216	5.947
Clinic vs. Institute	1.251	4.328	0.037	3.495	1.075	11.359

OR, odds ratio; 95% CI, 95% Confidence Interval; *B*, regression coefficient.
